# A “cluster” of ten uterine anomalies observed in a single center over a short period of 4 weeks: a case series

**DOI:** 10.1186/s13256-022-03362-2

**Published:** 2022-04-02

**Authors:** Yogindrakumar M. Kabadi, Abirami Ayyanar

**Affiliations:** 1 Dept of obstetrics and gynaecology, Karnataka Institute Of Medical Sciences, Block 12/2 G O Quarters KIMS vidyanagar, Hubballi, 580022 Karnataka India; 2 Department of Obstetrics and Gynaecology, Karnataka Institute of Medical Sciences (KIMS), Hubli, Karnataka India

**Keywords:** Uterine anomalies, Cluster of cases, Intraoperative diagnosis, Bicornuate uterus, Unicornuate uterus, Case series

## Abstract

**Background:**

Uterine anomalies occur because of Müllerian duct maldevelopment. Few of them are associated with adverse obstetric outcome (Reyes-Muñoz *et al.* in Diagnostics. 2019;9:4. https://doi.org/10.3390/diagnostics9040149). Genital outflow tract obstructive uterine anomalies invariably present in the adolescent age group.

**Case details:**

We report a case series of uterine anomalies. Ten such cases presented like a “cluster” within a short span of just one month. Eight of these ten cases were diagnosed intraoperatively during cesarean section. One case was diagnosed during laparoscopic sterilization, and the other case was diagnosed before doing manual vacuum aspiration. There were four cases of bicornuate uterus, two cases each of unicornuate uterus and uterine didelphys, and one case each of septate uterus and arcuate uterus. All eight babies were healthy and without any obvious congenital anomalies. To the best of the authors’ knowledge, literature regarding these anomalies has been mentioned mostly as case reports (Bruand *et al.* in Cureus. 2020;12:3. https://doi.org/10.7759/cureus.7191) and a few case series (Ross *et al.* in BMJ Case Rep. 2018. https://doi.org/10.1136/bcr-2017-221815). All women were of Kannadiga ethnicity and in the age range of 19–35 years. They were from places nearby to our institute within a range of approximately 250 km.

**Conclusion:**

We describe herein almost all types of uterine anomalies. These rare uterine anomalies presented in a short span of just four weeks like a “cluster”. This incidental finding is unusual. We need to design studies to understand the reasons for clustering of such cases in our clinical practice.

## Introduction

Uterine malformations are as result of agenesis, hypoplasia, abnormal fusion, or resorption of Müllerian ducts. The causes of uterine malformations are multifactorial. Some of them are ionizing radiations, viral infections, drug induced and genetic mutation involving *BCL2* gene. The incidence of Müllerian anomaly is 0.2%–3.8% in fertile women and 3.5%–6.3% in infertile women. In one study, it was 4.4%, with septate uterus leading the anomalies [1]. Women with uterine malformations who are asymptomatic and are not operated remain undiagnosed. Some of the anomalies in these women may be diagnosed during imaging, Lower segment cesarean section (LSCS), laparotomy, or laparoscopy, as in our last case (Table 1). Müllerian duct anomalies are classified according to the 1988 American Fertility Society classification. We mention here the number of cases in our series in each category of the classification.

**Table 1:** Clinical data and management of index cases

Case no.	Age (years)	Clinical presentation	Menstrual history	Obstetric history	Clinical examination findings	Antenatal USG	Indication for LSCS	Status of the baby	Intraoperative diagnosis
**1.**	22	G6A5, 34 weeks GA with recurrent pregnancy loss with APE	Regular	Married life 7 years, G6A5, all being spontaneous abortion during first trimester	not contributary	normal findings	APE	No anomalies	Septate uterus
**2.**	20	G2A1, 39 weeks and 6 days GA with breech presentation not in labor	Regular	G2A1, first pregnancy with spontaneous abortion during first trimester	not contributary	normal findings	Breech presentation	No anomalies	Arcuate uterus
**3.**	19	Primigravida with 9 weeks GA with inevitable abortion	Regular	primigravida	Per speculum showed septum with two vaginae and two cervices on either side	9 weeks gestation with uterine didelphys	N/A	N/A	Uterine didelphys diagnosed during ultrasonography
**4.**	20	G2P1L1, 38 weeks +6 days GA with previous LSCS in latent labor with uterine didelphys	Regular	Married life 3 years, first baby healthy, LSCS done for breech presentation	Per speculum showed septum with two vaginae and two cervices on either side	normal findings	Previous LSCS, with threatened scar rupture	No anomalies	Uterine didelphys with longitudinal septum found during LSCS
**5.**	20	G3A2, 35 weeks + 4 days GA with severe preeclampsia with ascites with breech presentation	Regular	Married life 5 years, G3A2, previous pregnancies all with first-trimester spontaneous abortion	not contributary	normal findings	Preterm breech with FGR with severe OH with breech	No anomalies	Bicornuate uterus
**6.**	25	Primigravida, 39 weeks + 5 days GA with severe preeclampsia with high leak > 8 hours not in labor	Regular	Primigravida, married life 1 year	Per vaginal examination in first trimester showed two horns with space in between (appreciated retrospectively)	SLIUG with average gestational age of 7 weeks + 6 days with bicornuate uterus	Primigravida with CPD in labor	No anomalies	Bicornuate uterus communicating cavity, pregnancy in right horn
**7.**	30	G2A1, 34 weeks + 5 days GA with cervical fibroid with footling presentation, moderate OH in active labor	Regular	G2A1, first pregnancy with spontaneous abortion during first trimester	Per vaginal examination in first trimester showed two horns with space in between (appreciated retrospectively)	SLIUG with average gestational age of 12 weeks with bicornuate uterus	Footling presentation	No anomalies	Bicornuate uterus
**8.**	35	G3P2L2 with 39 weeks + 2 days GA with two previous LSCS with severe preeclampsia, high leak > 2 hours not in labor	Regular	G3P2L2, married life 10 years. First and second pregnancy uneventful	not contributary	normal findings	Previous two LSCS with severe preeclampsia with high leak	No anomalies	Unicornuate uterus
**9.**	30	G2P1L1 40 weeks + 4 days GA with previous LSCS with H/O cardiac surgery, not in labor	Regular	G2P1L1, first baby healthy and LSCS done for CPD	not contributary	normal findings	Previous LSCS with threatened scar rupture	No anomalies	Unicornuate uterus
**10.**	28	P2L2 for laparoscopic sterilization	Menarche at 13 years, regular	Married for 7 years. Both previous pregnancies uneventful	not contributary	N/A	N/A	N/A	Bicornuate uterus with rudimentary left horn

*GA* gestational age, *APE* antepartum eclampsia, *MVA* manual vacuum aspiration, *LSCS* lower-segment cesarean section, *FGR* fetal growth retardation, *OH* oligohydramnios, *CPD *cephalopelvic disproportion, *H/O *history of, *N/A* not applicable, *SLIUG* single live intrauterine gestation, normal findings - is with respect to uterine anomalies only

The number of cases in each group is as follows. Class I: agenesis/hypoplasia (nil); class II: unicornuate (two cases); class III: didelphys (two cases); class IV: bicornuate (four cases); class V: septate (one case); class VI: arcuate (one case); class VII: drug induced (nil).

As per our thorough literature search, most of these anomalies have been reported as isolated case reports [2], while case series mention a very small number of cases with anomalies [3]. Our series of ten cases presenting in a span of just one  month is like a “cluster”. In this cluster, we were able to identify almost all the uterine malformations. Eight out of the ten cases were diagnosed intraoperatively during LSCS/laparoscopy. In epidemiology, a cluster is defined as an aggregation of cases of a disease or a health-related condition, such as a cancer or birth defect, closely grouped in time and place. Here, we could not find any geographic or demographic similarities among this cluster of women with uterine malformations. We therefore call it a cluster, although it does not fit the exact definition. Yet in our practice, we see clustering of similar types of cases especially in the labor room, which cannot be explained by anything but chance occurrence. But, by attributing it to chance occurrence, are we missing something that needs to be understood or discovered? Appropriate studies need to be planned and carried out to determine the cause of such occurrence of events in clusters, which at present appear to be just due to chance.

## Case details

A total of ten cases admitted to the Department of Obstetrics and Gynaecology during November and December 2020 in a tertiary care hospital in South India had various uterine malformations. For all these cases written informed consent for the specific intervention of surgery and anesthesia was taken. Consent for photographing/recording of events and for publications (if done) was also taken, and patients consented to the same. Institutional review board approval of the Medical Research Unit of our institute (Karnataka Institute Of Medical Sciences Hubballi) was taken. Eight cases admitted near term underwent LSCS . One case with 9 weeks of gestation underwent manual vacuum aspiration (MVA). One case was admitted for laparoscopic sterilization. The demographic and clinical data are presented in Table 1. All women were of Kannadiga ethnicity and in the age range of 19–35 years. They were from places nearby to our institute within a range of approximately 250 km.

The mean age was 24.7 years (median 23.5 years, range 19–35 years). Eight cases presented in the third trimester. One case presented at 9 weeks for MVA, and another case was admitted for laparoscopic sterilization. Median gestational age was 38 weeks, ranging from 9 to 40 weeks. Four of these cases had history of spontaneous abortion in first trimester. There is no history of interventions for uterine anomaly correction before pregnancy in any of these cases. None of these cases had past history of known uterine anomalies (they were undiagnosed/ or intraoperative history not available). None had any past history of diabetes, hypertension, endocrine disorders, or Anti Phospholipid Antibody Syndrome (APLA) syndrome. One of the women had undergone cardiac surgery in the past. Family history of any other congenital anomalies was absent. There was no other significant family history. Menstrual history was regular and uneventful in all cases. The indications for LSCS in these cases were antepartum eclampsia, breech presentation, previous LSCS with threatened scar rupture, fetal growth retardation, oligohydramnios, cephalopelvic disproportion (CPD), footling presentation, and preterm breech as shown in the table.

**Fig. 1:**
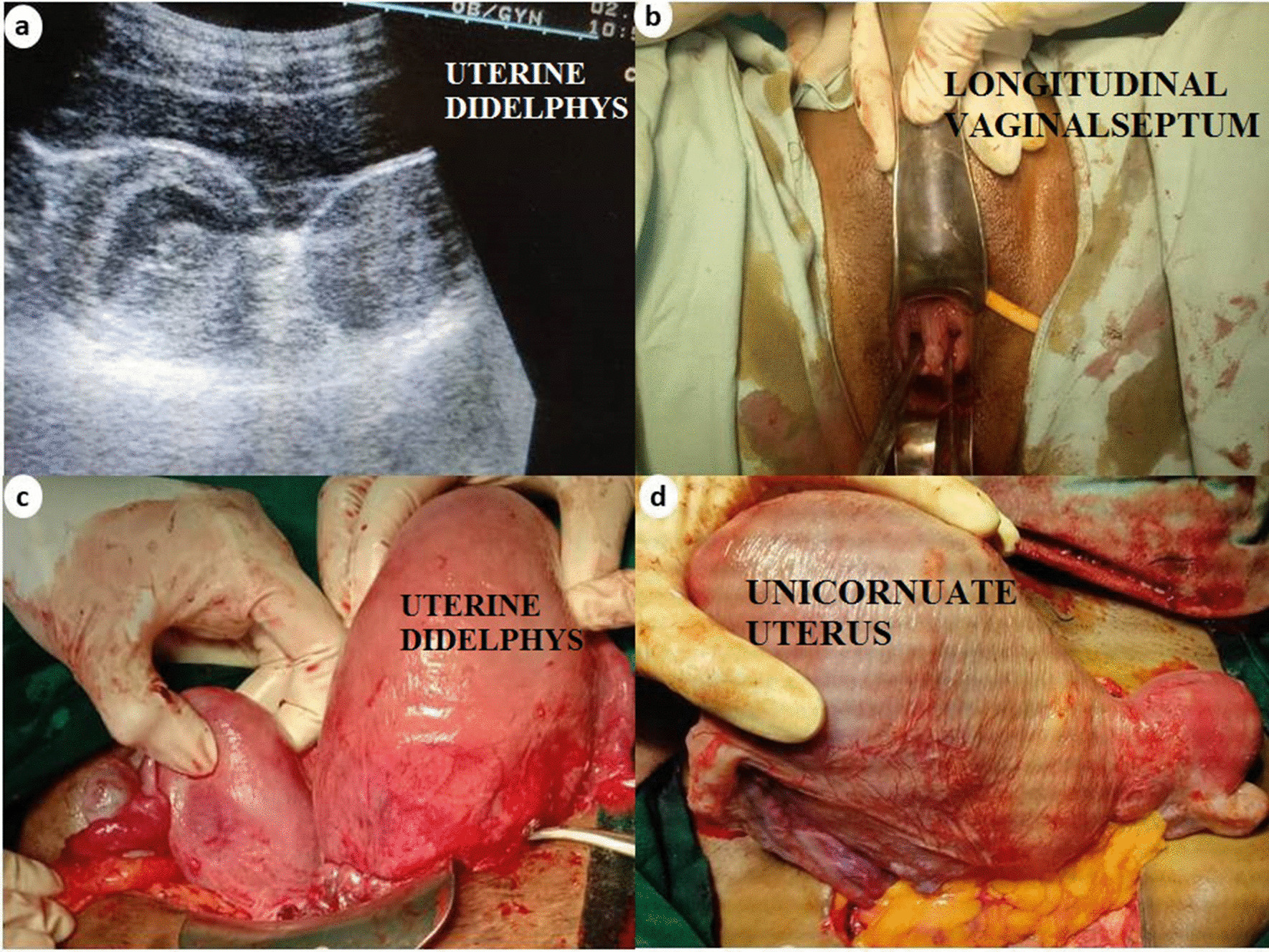
**a** USG image of uterine didelphys (USG image) and **b** longitudinal vaginal septum (on per speculum examination) in the same patient; **c** intraoperative image of uterine didelphys; **d** intraoperative image of unicornuate uterus

**Fig. 2:**
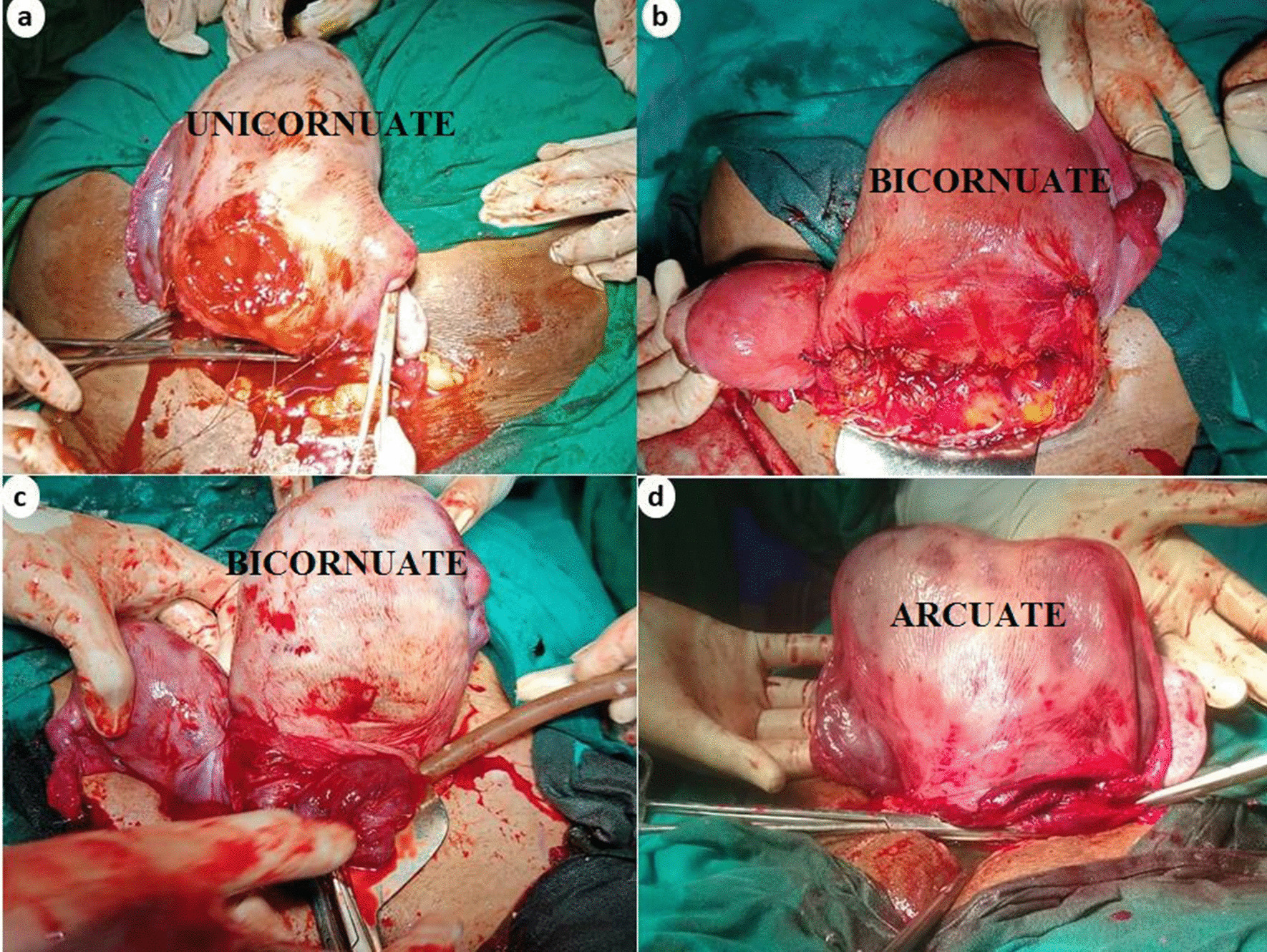
Intraoperative images of **a** unicornuate, **b** bicornuate, **c** bicornuate, and **d** arcuate uterus

Antenatal diagnosis of uterine malformation by USG was available in only two cases. One case showed single live intrauterine gestation (SLIUG) of 7 weeks + 6 days with bicornuate uterus (Table 1, case 6) while another case (Table 1, case 3) showed SLIUG of 9 weeks with uterine didelphys. Per vaginal examination findings of bicornuate uterus (case 6) were appreciated only after first trimester ultrasonography diagnoses was available. The rest of the cases were diagnosed intraoperatively during LSCS (seven cases) and one during laparoscopic sterilization. The significant antenatal details of all the cases is summarized in the table. Figures 1 and 2 show photographs of the cases. All these cases did not have any problems during their stay in the hospital. Eight cases who delivered had healthy babies without any obvious congenital anomalies.

## Discussion

Müllerian ducts are the primordial anlage of the female reproductive tract that differentiates to form the fallopian tubes, body of uterus, cervix, and the upper part of vagina. Müllerian duct anomalies may lead to adverse obstetric outcome.

The mean age of presentation is typically in adolescence for girls with genital tract outflow obstructive anomalies. In contrast, the mean age of presentation in our series as obstetric cases is 24.7 years, which is comparable to studies [4], showing slightly higher age, which can be explained by the few who suffered poor obstetric history. Due to the close embryologic relationship between paramesonephric and mesonephric ducts, failure of development in both of them may result in the coexistence of congenital uterine and renal anomalies. Congenital ipsilateral renal agenesis was reported in literature in 16–38% of women with unicornuate uterus. None of our cases had associated renal congenital anomalies. USG helps early identification of uterine anomalies. One of the previous case series mentioned that four out of seven cases were diagnosed by USG [4]. In contrast, we had only two cases with USG diagnosis of uterine anomalies in the antenatal period. Intraoperative diagnosis during cesarean section was reported in three out of seven cases [4]. Similarly, in the present study, eight out of ten cases were diagnosed during caesarean section or laparoscopic sterilization. In our study, most of the cases were diagnosed intraoperatively and not antenatally could be explained due to difficulty or inability to diagnose antenatally.

As described in many studies, uterine anomalies result in adverse obstetric outcomes including early pregnancy loss, malpresentation, and preterm labor. These adverse effects are related to specific types of uterine anomalies. Among these adverse outcomes, malpresentation (predominantly breech presentation) are common and lead to cesarean delivery. It has been reported that rates of cesarean delivery are increased in cases of uterine anomalies. This finding is similar to our case series, in which 80% of cases were diagnosed intraoperatively during LSCS.

Such clinical presentation of uterine anomalies as a cluster and within a short period has not been described previously. Our case series included almost all uterine anomalies in a significant number, presented in a short span of time like a cluster at a single institute. This is an unusual occurrence. This might be a rare incidental finding worth mentioning as a rare cluster of uterine anomalies. We need to devise studies to study the cause of such clustering of cases that appear like chance occurrences.

## Conclusion

The present case series describes almost all types of uterine anomalies within a very short span of four weeks like a cluster (whereas it usually takes more time to accumulate such a number of cases) [4], and most of them were diagnosed intraoperatively. Diagnosis of uterine malformations is usually missed, but they should be considered in cases of poor obstetric history or women presenting with infertility. Quite a number of times, we have found noninfectious events of a particular nature occurring repeatedly over a period of time in our clinical practice, which we attribute to chance occurrence. We need to identify such clustering and formulate studies to determine the reason for such events in our clinical practice. This would be useful if the events are life threatening or need prior preparation.

## Data Availability

Supporting data in the source document and study file are available and can be provided if necessary.
